# A retrospective study assessing the clinical outcomes and costs of acute hepatitis A in Cape Town, South Africa

**DOI:** 10.1186/s12879-021-06993-w

**Published:** 2022-01-11

**Authors:** Jenna Patterson, Susan Cleary, Sheetal P. Silal, Gregory D. Hussey, Annabel Enoch, Stephen Korsman, Elizabeth Goddard, Mashiko Setshedi, Wendy C. Spearman, Benjamin M. Kagina, Rudzani Muloiwa

**Affiliations:** 1 Division of Epidemiology and Biostatistics, School of Public Health and Family Medicine, University of Cape Town, Cape Town, South Africa; 2 Vaccines for Africa Initiative, School of Public Health and Family Medicine, University of Cape Town, Cape Town, South Africa; 3 Division of Health Economics, School of Public Health and Family Medicine, University of Cape Town, Cape Town, South Africa; 4 Modelling and Simulatio Hub Africa, Department of Statistical Sciences, University of Cape Town, Cape Town, South Africa; 5 Division of Medical Microbiology, Institute of Infectious Disease and Molecular Medicine, University of Cape Town, Cape Town, South Africa; 6 National Health Laboratory Service, Groote Schuur Hospital, Cape Town, South Africa; 7 Division of Medical Virology, Department of Pathology, University of Cape Town, Cape Town, South Africa; 8 Department of Paediatrics and Child Health, Red Cross War Memorial Children’s Hospital, University of Cape Town, Cape Town, South Africa; 9 Department of Medicine, Groote Schuur Hospital, University of Cape Town, Cape Town, South Africa

**Keywords:** Epidemiology, Health economics, Vaccine preventable disease, Immunization, Hepatology, Hepatitis A, Acute liver failure

## Abstract

**Background:**

While some evidence has been demonstrated the cost-effectiveness of routine hepatitis A vaccination in middle-income countries, the evidence is still limited in other settings including in South Africa. Given this, the evidence base around the cost of care for hepatitis A needs to be developed towards considerations of introducing hepatitis A vaccines in the national immunisation schedule and guidelines.

**Objectives:**

To describe the severity, clinical outcomes, and cost of hepatitis A cases presenting to two tertiary healthcare centers in Cape Town, South Africa.

**Methods:**

We conducted a retrospective folder review of patients presenting with hepatitis A at two tertiary level hospitals providing care for urban communities of metropolitan Cape Town, South Africa. Patients included in this folder review tested positive for hepatitis A immunoglobulin M between 1 January 2008 and 1 March 2018.

**Results:**

In total, 239 folders of hepatitis A paediatric patients < 15 years old and 212 folders of hepatitis A adult patients $$\ge$$ 15 years old were included in the study. Before presenting for tertiary level care, more than half of patients presented for an initial consultation at either a community clinic or general physician. The mean length of hospital stay was 7.45 days for adult patients and 3.11 days for paediatric patients. Three adult patients in the study population died as a result of hepatitis A infection and 29 developed complicated hepatitis A. One paediatric patient in the study population died as a result of hepatitis A infection and 27 developed complicated hepatitis A, including 4 paediatric patients diagnosed with acute liver failure. The total cost per hepatitis A hospitalisation was $1935.41 for adult patients and $563.06 for paediatric patients, with overhead costs dictated by the length of stay being the largest cost driver.

**Conclusion:**

More than 1 in every 10 hepatitis A cases (13.3%) included in this study developed complicated hepatitis A or resulted in death. Given the severity of clinical outcomes and high costs associated with hepatitis A hospitalisation, it is important to consider the introduction of hepatitis A immunisation in the public sector in South Africa to potentially avert future morbidity, mortality, and healthcare spending.

**Supplementary Information:**

The online version contains supplementary material available at 10.1186/s12879-021-06993-w.

## Background

The epidemiology of hepatitis A remains unclear globally. The World Health Organization (WHO) describes the epidemiology of hepatitis A according to hepatitis A virus (HAV) endemicity levels measured by the proportion of people with anti-HAV Immunoglobulin G (IgG) antibodies [1]. In areas where there is high exposure to the virus (high HAV endemicity), a large percentage of the population is assumed to have been asymptomatically infected by 10 years old [1]. Due to improvements in water, sanitation, and developments in socioeconomic status, low- and middle-income countries may transition from high to intermediate or low HAV endemicity. In areas of intermediate or low HAV endemicity, a lower proportion of the respective populations will have been infected during childhood, and the likelihood of symptomatic infection during adulthood increases [2]. In these cases, WHO recommends the consideration of introducing hepatitis A vaccines to reduce morbidity and mortality due to the disease [1].

Since 2005, there has been a documented shift in hepatitis A epidemiology in South Africa with a rise in the number of clinically symptomatic hepatitis A cases indicated by high anti-HAV Immunoglobulin M (IgM) positivity rates, especially among children and adolescents < 15 years old [2–5]. Analyses of routine HAV laboratory data between 2005 and 2015 in South Africa suggest that children < 5 years old carry the largest burden of acute hepatitis A compared to other age groups. Additionally, these analyses point out that the seroprevalence of anti-HAV reaches levels > 90% only in individuals > 25 years old, suggesting South Africa should be classified as a country with intermediate HAV endemicity.

Despite South Africa’s intermediate HAV endemicity status, hepatitis A vaccines are not currently included in the National Expanded Programme on Immunisation (EPI) even as the cost-effectiveness of universal hepatitis A vaccination is well documented in low and intermediate HAV endemicity regions such as Argentina, Brazil, Chile, and Mexico [6–8]. It has, therefore, become important to consider the local hepatitis A morbidity, mortality, and costs of care to bolster considerations of introducing the vaccine in South Africa.

## Methods

### Aim

The aim of this study is to describe the clinical severity and costs of care for hepatitis A cases presenting to two public sector tertiary healthcare centers in Cape Town, South Africa. The results of this study will be used together with other ongoing research to forecast the health impacts and cost-effectiveness of different hepatitis A vaccination strategies to be considered for inclusion in the EPI.

### Setting and participants

We conducted a retrospective folder review of patients presenting with hepatitis A to two tertiary level hospitals providing care for urban communities of metropolitan Cape Town, South Africa. The hospitals included Red Cross War Memorial Children’s Hospital (RCH) serving paediatric patients < 15 years old and Groote Schuur Hospital (GSH) serving adult patients $\ge$ 15 years old.

Patients included in this folder review were identified by flagging all positive hepatitis A immunoglobulin M (IgM) tests between 1 January 2008 and 1 March 2018 through the South African National Health Laboratory Services database. Once folder numbers corresponding to positive IgM tests were identified, patient folders were reviewed for inclusion eligibility. Patients with clinically confirmed hepatitis A and without evidence of concomitant hepatitis E infection were selected for inclusion in this study.

### Hepatitis A case definition

Included cases needed to meet both the clinical description and laboratory confirmation of acute hepatitis A, as defined by the Centres for Disease Control and Prevention (CDC) [9].

- Clinical description: An acute illness with a discrete onset of any sign or symptom consistent with acute viral hepatitis and either a) jaundice, or b) elevated serum alanine aminotransferase (ALT) or aspartate aminotransferase (AST) levels. Symptoms of acute viral hepatitis include fever, headache, malaise, anorexia, nausea, vomiting, diarrhoea, and abdominal pain.
- Laboratory criteria: Positive sera identification of immunoglobulin M (IgM) antibody to the hepatitis A virus.

At the time of admission, evidence of acute liver injury was assessed through analysis of international normalised ratio (INR), elevated levels of alanine aminotransferase (ALT), aspartate aminotransferase (AST), alkaline phosphatase (ALP), and total bilirubin [10, 11]. Patient clinical outcomes were classified as uncomplicated, complicated, or deceased within further analysis.

### Hepatitis A outcome definitions

Complications included the development of relapsing hepatitis, prolonged cholestasis, acute liver failure, or had comorbidities that complicated care and recovery.

- Relapsing hepatitis A was defined in all patients as being re-admitted for hepatitis A within 6 months of first admission.
- Prolonged cholestasis was defined in all patients with prolonged jaundice lasting longer than 14 days and conjugated bilirubin > 10 IU/L.
- Acute liver failure in paediatric patients was defined as INR ≥ 1.5 not corrected by vitamin K in the presence of clinical hepatic encephalopathy or INR ≥ 2.0 regardless of the presence or absence of clinical hepatic encephalopathy [10, 12].
- Acute liver failure in adult patients was defined as hepatic encephalopathy and coagulopathy INR ≥ 1.5, in patients without pre-existing cirrhosis, and an illness of < 26 weeks duration [13].

### Data collection

Data were extracted from folders and corresponding electronic records by a clinical registrar using a pre-designed piloted data extraction form. The data extraction form was piloted using 50 patient folders before the start of the study. The following data elements were included in the data extraction form: demographic information, hepatitis A risk factors, hospital admission and discharge dates, clinical signs and symptoms of hepatitis A, bloods drawn, medicines and products used for case management, medicines prescribed at discharge, clinical outcome, and length of stay in varying hospital wards. In addition, data on the patient account were extracted from the folders to estimate the percentage of the cost of hepatitis A cases carried by the national governments as the ultimate fee payer for the included facilities.

During data extraction, study IDs were generated to identify patients so that names and/or any patient identifying information were not included in the data extraction process. Data were analysed in STATA version 16.0 [14]. Clinical characteristics, demographics, and clinical variables were summarised using descriptive statistics. Means, medians, and interquartile ranges were calculated for continuous variables, while counts and percentages were calculated for categorical variables.

All study data were subsequently removed from Kobo Toolbox and have been saved on a password-protected hard drive which will be kept by the first author for 5 years. The study was approved by the University of Cape Town's Research Ethics Committee and research clearance was granted by GSH (HREC 485) and RCH’s Research Committees (RCC 153).

### Costing

Costing for hepatitis A cases was conducted following recommendations for conducting and reporting of economic evaluations as per the CHEERS guidelines [15]. To estimate the cost of care for hepatitis A cases, this study calculated the mean cost per hepatitis A hospitalisation from a health care provider's perspective. Costing of hepatitis A cases entailed the multiplication of counts of health service utilisation against unit costs. Counts of service utilisation were achieved using data extracted from folders. Thereafter, unit costs were established using a combination of the ingredients and step-down methods, as appropriate. In this process, the ingredients approach was first applied to cost the items directly used for the diagnosis and treatment of hepatitis A cases, including laboratory investigations, procedures, medications, and blood products [16]. Laboratory and blood unit costs were provided by the National Health Laboratory Services. Medicine unit costs were obtained from the 2018 National Tender Price List [17]. Radiology and other imaging investigation costs were derived from the 2018 National Uniform Patient Fee Schedule [18].

Overhead resources that could not be costed from the folder review were costed using the step-down approach. In the step-down approach, overhead expenditures were established from facility accounting records and were allocated to routine patient activity data to establish an overhead cost per inpatient day.

All costs were expressed in September 2018 prices and converted to US dollars using an average exchange rate over the same period (US$1 = 14.75 South African Rand) [19]. One-way deterministic sensitivity analyses were conducted on the three largest patient-specific cost components including blood tests, medicines, and radiology tests to explore the impact on the mean cost per hepatitis A hospitalization. Each component was varied using the 90% CI and the results are displayed in tornado diagrams.

## Results

### Demographics and risk factor characteristics of the study population

In total, 239 folders of hepatitis A paediatric patients < 15 years old (median = 6.6 years old) and 212 folders of hepatitis A adult patients $\ge$ 15 years old (median = 27.4 years old) were included in the study. A total of 8 adult patients and 6 paediatric patients included in the study were confirmed as HIV positive. Five adult patients and one paediatric patient were also positive for hepatitis B surface antigen (HBs-Ag), while one adult patient was also positive for hepatitis C. For patients where this information was reported, regular use of toilets without plumbing and sharing of communal taps were the most frequent hepatitis A risk factors among both adult and paediatric patients. The demographic and risk factor characteristics of the patients are further summarised in Table 1.

**Table 1:** Demographics and risk factors for hepatitis A patients among the study patient population

Variable	Number of adult patients (%)	Number of paediatric patients (%)
Number of patients	212	239
Median age (interquartile range)	27.4 (21.5, 34.3)	6.6 (4.4, 8.9)
Gender		
Female	94 (44.3%)	125 (52.3%)
Male	118 (55.7%)	114 (47.7%)
Patient account class		
Patient pays nominal fees	169 (79.7%)	194 (81.2%)
Patient pays a portion of fees	12 (5.7%)	24 (10.0%)
Patient pays fees in full	22 (10.4%)	14 (5.9%)
Not recorded	9 (4.2%)	7 (2.9%)
Known contact with hepatitis A case	20 (9.4%)	16 (6.7%)
Housing		
Informal housing	9 (4.3%)	9 (3.8%)
Formal housing	9 (4.3%)	48 (20.1%)
Housing type not reported	194 (91.5%)	182 (76.1%)
Water source		
Water source from outside dwelling	11 (5.2%)	58 (24.3%)
Water source not reported	201 (94.8%)	181 (75.7%)
Sanitation		
Toilet without plumbing	19 (9.0%)	56 (23.44%)
Toilet type not reported	193 (91.0%)	183 (76.6%)
Additional risk factors*		
Alcohol use	46 (21.7%)	–
IV drug use	20 (9.4%)	–
Travel history	10 (4.7%)	7 (3.0%)

All variables are presented as N (%)

^*^Additional risk factor information including alcohol and IV drug use was not collected for paediatric patients

### Clinical presentation and severity

Clinical signs and symptoms of hepatitis A at the time of admission are summarised in Table 2. More than half of patients in the study reported vomiting and abdominal pain as symptoms of hepatitis A before presentation for care at respective facilities. More than half of adult patients presented for care with clinical signs of jaundice, while more than half of paediatric patients presented for care with clinical signs of jaundice and enlargement of the liver. As displayed in Table 3, > 80% of adult patients and > 90% of paediatric patients who developed complicated hepatitis A displayed evidence of acute liver injury at the time of admission. All patients who died from hepatitis A infection displayed evidence of prolonged INR at the time of admission.

**Table 2:** Hepatitis A clinical presentation

Variable	Number of adult patients (%)	Number of paediatric patients (%)
Prevalence of clinical symptoms		
Abdominal pain	110 (51.9%)	121 (50.6%)
Anorexia	58 (27.4%)	90 (37.7%)
Dark urine	83 (39.2%)	105 (43.9%)
Diarrhea	26 (12.3%)	62 (25.9%)
Drowsiness	4 (1.9%)	16 (6.7%)
Fatigue	49 (23.1%)	37 (15.5%)
Fever ≥ $38^\circ{\rm C}$	68 (32.1%)	71 (29.7%)
Headache	21 (9.9%)	15 (6.3%)
Jaundice	155 (73.1%)	198 (82.9%)
Joint ache	8 (3.8%)	0 (0.0%)
Nausea	84 (39.6%)	20 (8.4%)
Pale stool	15 (7.1%)	13 (5.4%)
Pruritis	103 (48.6%)	58 (24.3%)
Respiratory symptoms	10 (4.7%)	22 (9.2%)
Vomiting	140 (66.0%)	166 (69.5%)
Prevalence of clinical signs		
Enlarged liver	60 (28.3%)	137 (57.3%)
Upper-right abdominal tenderness	104 (49.1%)	105 (43.9%)

**Table 3:** Hepatitis A clinical outcomes and evidence of acute liver injury

Adult patients				
Evidence of acute liver injury	All patients (N = 212)	Uncomplicated hepatitis (n = 180, 84.9%)	Complicated hepatitis A (n = 29, 13.7%)	Deceased (n = 3, 1.4%)
INR ≥ 1.5	12 (5.6%)	9 (5.0%)	0 (0.0%)	3 (100.0%)
ALT > 40 U/L	199 (93.9%)	168 (93.3%)	27 (93.1%)	3 (100.0%)
AST > 40 U/L	197 (92.9%)	168 (93.3%)	26 (89.7%)	3 (100.0%)
ALP > 128 U/L	192 (90.6%)	165 (91.7%)	24 (82.8%)	3 (100.0%)
Total bilirubin > 21 U/L	188 (88.7%)	160 (88.9%)	25 (86.2%)	3 (100.0%)

Paediatric patients				
Evidence of acute liver injury	All patients (N = 239)	Uncomplicated hepatitis (n = 211, 88.3%)	Complicated hepatitis A (n = 27, 12.8%)	Deceased (n = 1, 0.4%)
INR 1.50–1.99	9 (3.8%)	8 (3.8%)	1 (3.7%)	0 (0.0%)
INR ≥ 2.0	4 (1.7%)	0 (0.0%)	3 (11.1%)	1 (100.0%)
ALT > 40 U/L	239 (100.0%)	211 (100.0%)	27 (100.0%)	1 (100.0%)
AST > 40 U/L	238 (99.6%)	211 (100.0%)	26 (96.3%)	1 (100.0%)
ALP > 128 U/L	233 (97.5%)	207 (98.1%)	25 (92.6%)	1 (100.0%)
Total bilirubin > 21 U/L	232 (97.1%)	206 (97.6%)	25 (92.6%)	1 (100.0%)

All variables are presented as N (%)

*ALT* alanine aminotransferase, *AST* alanine aminotransferase, *U/L* units per liter

### Clinical outcomes

Among the study population, 4 patients (0.9%) died as a result of hepatitis A infection [GSH 3/212 (1.4%); RCH 1/239 (0.4%)]. In addition to these deaths, 56 patients (12.4%) developed complicated hepatitis A [GSH 29/212 (13.7%); RCH 27/239 (11.3%)]. Of the 14 HIV + patients included in the study, 7 of these patients (50%) developed complicated hepatitis. One adult patient and two paediatric patients developed relapsing hepatitis A. Four paediatric patients developed prolonged cholestasis. Four paediatric patients and one adult patient included in this study developed acute liver failure from hepatitis A infection. One of these paediatric acute liver failure patients died during hospitalisation, however, no ALF patients in the study population were sent for transplant.

### Hepatitis A patient clinical service utilisation

**Fig. 1:**
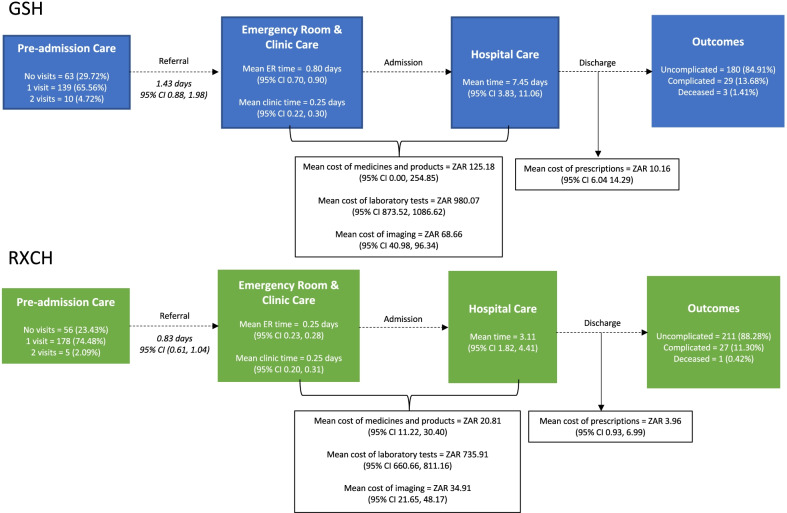
Hepatitis A patient facility utilization and clinical outcomes

**Fig. 2:**
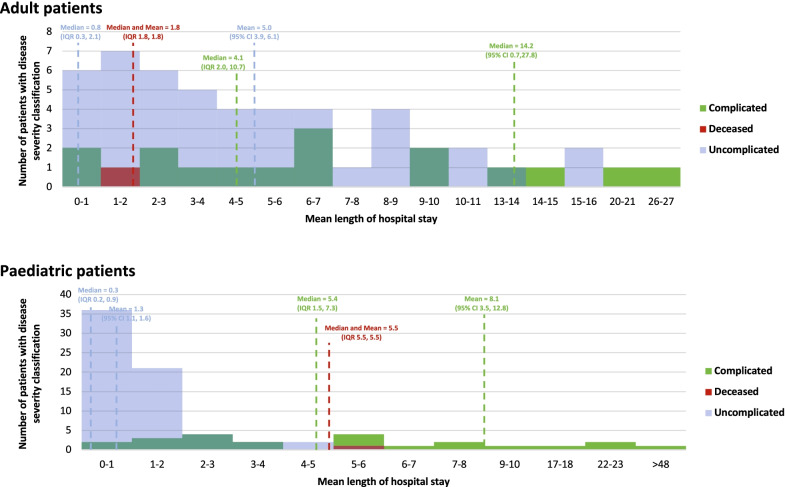
Length of hospitalization by patient outcome

Before presenting for tertiary level care, 332 patients (73.6%) [GSH 149/212 (70.3%); RCH 183/239 (76.6%)] presented for an initial consult at either a community health center, clinic, or general physician (Fig. 1). Following presentation for care at the primary level, the mean time for adults to present to GSH tertiary clinic or emergency room was 34.3 h (median = 17.1 h) and the mean time for paediatric patients to present to RCH tertiary level clinic or emergency room was 20.0 h (median = 17.0 h). The mean times spent in the emergency room or outpatient consulting ro in the teritiary hospital prior to admission for adult patients were 6 h (median = 6.2 h) and 19.2 h (median = 6.2 h), respectively. The mean times spent in the tertiary facility clinic and emergency rooms before hospital admission for paediatric patients were 6 h (median = 5.5 h) and 6 h (median = 4.7 h), respectively. The mean length of inpatient hospital stay was 7.45 days (median = 0.8 days) for adult patients and 3.11 days (median = 0.3 days) for paediatric patients, which was largely influenced by the clinical outcome as displayed in Fig. 2 and Additional file 1: Table S1.

### Hepatitis A costing

Using 2018 financial reports for each of the included facilities, overhead costs are presented in Table 4. Disaggregated expenditures in Table 4 and the patient volumes reported per facility in 2018 yielded a cost per patient day equivalent of $249.00 for GSH and $163.71 for RCH.

**Table 4:** Cost per patient day equivalent at included facilities in USD

Overhead line item	Groote Schuur Hospital serving adult patients	Red Cross Children’s War Memorial Hospital serving paediatric patients
Compensation of employees	$119,892,542.40	$9,548,135.59
Employee benefits	$556,000.00	$175,457.63
Goods and services	$27,203,728.81	$9,441,423.73
Machinery and equipment	$1,715,050.85	$878,983.05
Software and intangible equipment	$16,949.15	$0.00
Total overhead costs	$149,384,271.20	$20,044,000.00
Total patient days	599,931	122,439
Overhead cost per patient day equivalent*	$249.00	$163.71

^*^To obtain the cost per patient day equivalent, the total overhead costs were divided by the total patient days per facility

The mean patient-specific hepatitis A costs including investigations, radiology, and medication were estimated to be $80.34 (95% CI 68.83, 91.86) for adult patients and $53.94 (95% CI 48.81, 59.07) for paediatric patients (Table 5). Additional file 2: Table S2 provides detail on the utilisation and unit costs of the investigations, radiology, and medicines and products. The most expensive blood and radiological tests conducted in these patient groups were antibody tests ($8.96) and gastroscopies ($74.92). The most expensive medicines and products were fresh frozen plasma for adult patients ($618.97) and prescriptions at discharge ($13.52) for paediatric patients.

**Table 5:** Hepatitis A patient-specific costs per hospitalisation at facilities in USD

Patient-specific cost	Mean adult cost in USD (95% CI)	Mean paediatric cost in USD(95% CI)
Laboratory tests	66.45 (59.2, 73.7)	49.89 (44.79, 55.0)
Radiology	4.65 (2.8, 6.5)	2.37 (1.47, 3.3)
Medications	9.24 (0.5, 18.0)	1.68 (0.99, 2.4)
Total	80.34 (68.8, 91.9)	53.94 (48.8, 59.1)

**Fig. 3:**
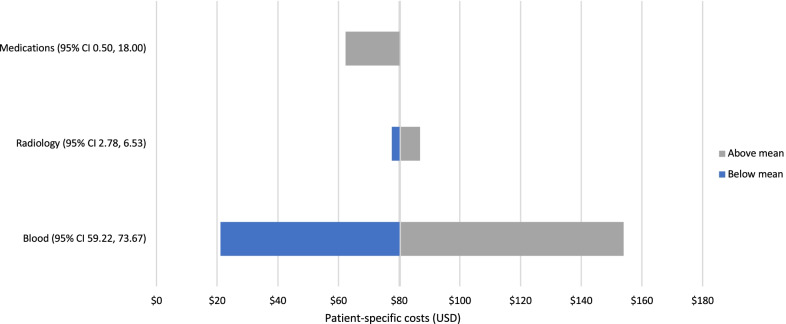
Sensitivity of patient-specific hepatitis A costs for adult cases

**Fig. 4:**
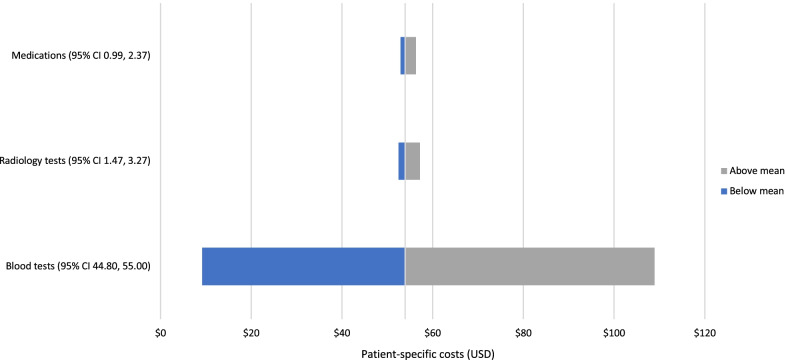
Sensitivity of patient-specific hepatitis A costs for paediatric cases

**Fig. 5:**
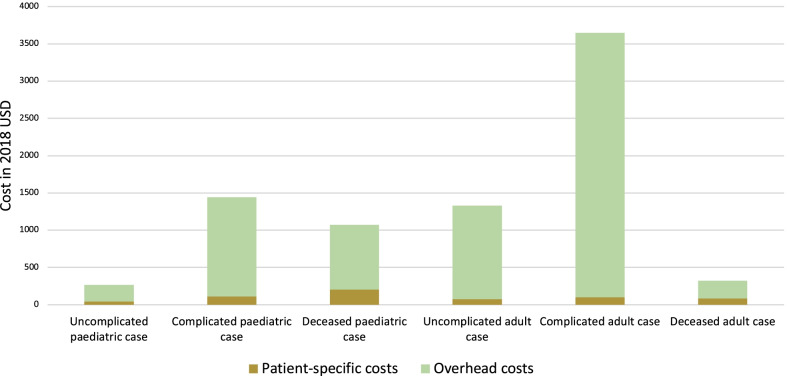
Components of total hepatitis A hospitalisation cost by patient clinical outcome

Using 95% CIs, sensitivity analyses were conducted to explore changes in the mean cost per admission associated with the three largest components of the patient-specific hepatitis A costs (blood tests, medicines, and radiology). Results are presented using tornado diagrams for adult and paediatric patients in Figs. 3 and 4, respectively. As displayed in these tornado plots, blood tests were the main cost driver for patient-specific hepatitis A costs in both adult and paediatric patients. Given the mean lengths of hospital stay for adult patients (7.45 days) and paediatric patients (3.11 days), the mean total cost per hepatitis A hospitalisation was $1935.41 for adult patients and $563.06 paediatric patients. The overhead costs dictated by the length of hospital stay were the main driver of total cost per hepatitis A hospitalisation as depicted in Fig. 5.

## Discussion

The results of this study indicate that hepatitis A causes severe disease in adults and children with 13.3% of the study population having suffered death (4 patients) or complication (56 patients). Of the patients who developed complications, almost all displayed evidence of acute liver injury at the time of admission. Of the patients who were HIV positive (14 patients), half developed complications due to hepatitis A infection. None of the acute liver failure patients included in this study population were referred for a liver transplant. To qualify for a transplant, social and socioeconomic criteria are used exclusion criteria for patients as transplant requires adherence to lifelong treatment and the presence of social support structures for positive outcomes.

The median length of hospital stay for hepatitis A cases included in this study was largely influenced by clinical outcomes. It is worrisome that patients who died under care had significantly shorter hospital stays meaning that these patients likely presented for care at a very late stage of infection. If patients did not die as a result of hepatitis A infection, they required significant resources for case management and treatment.

Blood tests were the main cost driver for patient-specific hepatitis A costs in both adult and paediatric patients, with an average of 3 blood panels ordered per hepatitis A case. The mean total cost per hepatitis A hospitalisation was $1,935.41 for adult patients and $563.06 for paediatric patients. The overhead costs dictated by the length of hospital stay were the main driver of total cost per hepatitis A hospitalisation. Notably, a large majority of adult and paediatric patients included in this study paid a nominal daily fee for hospitalisation and the government was responsible for paying > 90% of the cost of treatment in more than 75% of cases included in this study. Further work should include an analysis of the impact of hepatitis A hospitalisation costs on the national health budget.

At large, the patient-specific hepatitis A cost estimates presented in this study are likely underestimates of the true costs of care. This folder review was conducted at tertiary level facilities, therefore, the study was unable to capture costs incurred for care at the primary level, which was utilised by approximately 70% of patients before presenting for tertiary care. The folder review also did not capture costs at non-tertiary hospitals, where patients (particularly severe patients who died under care at GSH and RCH) may have sought care before presenting at the tertiary levels facilities included in this study. Additionally, the adoption of a provider’s perspective in this study led to the exclusion of costs incurred by patients for care and did not allow for the opportunity costs of accessing care including out-of-pocket payments and loss of productivity. Lastly, the folder review did not include additional costs of providing prophylaxis to close contacts of hepatitis A cases with HAV vaccine or immunoglobulin according to clinical guidelines in South Africa [20].

Additional limitations of this study include that the underlying epidemiological characteristics of the study population were not well described as comorbidities were not well documents and not all patients were tested for HIV, hepatitis B, or hepatitis C at the time of admission. Information on hepatitis A risk factors such as housing, sanitation, and water source were also not routinely reported in patient folders. Patients presenting to the included tertiary facilities cannot be considered representative of the general South African population, however, the clinical outcome and cost data collected in this study provides a better local context than is currently represented in published literature.

Notwithstanding the noted limitations, this is the first study to describe the clinical severity and costs of care for hepatitis A cases in South Africa. The study highlights the notable severity of hepatitis A infection experienced by many patients in South Africa and the high burden of cost on the national health budget for case management and treatment of the disease. This study is part of an ongoing body of work to determine the cost-effectiveness of introducing hepatitis A vaccines into the South African Expanded Programme on Immunization. The ongoing work includes a dynamic model to estimate the epidemiological and economic outcomes for different hepatitis A vaccination strategies in the country.

## Conclusion

More than 1 in 10 hepatitis A cases included in this study developed complicated hepatitis A or died as a result of infection. Given the severity of clinical outcomes and high costs associated with hepatitis A hospitalisation, it is important to consider the introduction of hepatitis A immunisation in the national immunisation program in South Africa to avert future morbidity, mortality, and significant healthcare spending from the national health budget.

### Risks and benefits

As this was a retrospective study, patient care was not affected. It is hoped that this study will help to highlight the significant burden that acute hepatitis A places on the population and will motivate for inclusion of the vaccine on a national scale.

## Supplementary Information

**Additional file 1: Table S1.** Mean and median lengths of hospitalisation by patient outcome.

**Additional file 2: Table S2.** Unit counts and cost (2018 USD) for patient-specific hepatitis A items.

## Data Availability

The datasets generated and/or analysed during the current study are not publicly available due to ongoing research but are available from the corresponding author upon reasonable request.

## Abbreviations

ALP: Alkaline phosphatase
ALT: Alanine aminotransferase
AST: Aspartate aminotransferase
CDC: Centres for Disease Control and Prevention
EPI: Expanded program on immunization
GSH: Groote Schuur Hospital
HAV: Hepatitis A virus
IgG: Immunoglobulin G
IgM: Immunoglobulin M
INR: International normalised ratio
RCH: Red Cross War Memorial Children’s Hospital
WHO: World Health Organization
