# Supplementary material for: A retrospective study assessing the clinical outcomes and costs of acute hepatitis A in Cape Town, South Africa
Source: BMC Infect Dis. 2022 Jan 11;22:45. doi: 10.1186/s12879-021-06993-w (PMC8751253; doi:10.1186/s12879-021-06993-w)
Supplement: Supplementary file 1 — Additional file 1: Table S1. Mean and median lengths of hospitalisation by patient outcome. [file 12879_2021_6993_MOESM1_ESM.docx]

| **Table S1: Mean and median lengths of hospitalisation by patient outcome** | | |
| --- | --- | --- |
| **Adult patients** | | |
| **Patient Outcome** | **Mean length of stay in days (95% CI)** | **Median length of stay in days (IQR)** |
| Uncomplicated (n=180) | 5.0 (3.9, 6.1) | 0.8 (0.3, 2.1) |
| Complicated (n=29) | 14.2 (0.7, 27.8) | 4.1 (2.0, 10.7) |
| Deceased (n=3) | 1.8 (1.8, 1.8) | 1.8 (1.8, 1.8) |
| **Paediatric patients** | | |
| **Patient Outcome** | **Mean length of stay in days (95% CI)** | **Median length of stay in days (IQR)** |
| Uncomplicated (n=211) | 1.3 (1.1, 1.6) | 0.3 (0.2, 0.9) |
| Complicated (n=27) | 8.1 (3.5, 12.8) | 5.4 (1.5, 7.3) |
| Deceased (n=1) | 5.3 (5.3, 5.3) | 5.5 (5.5, 5.5) |
