# Supplementary material for: A retrospective study assessing the clinical outcomes and costs of acute hepatitis A in Cape Town, South Africa
Source: BMC Infect Dis. 2022 Jan 11;22:45. doi: 10.1186/s12879-021-06993-w (PMC8751253; doi:10.1186/s12879-021-06993-w)
Supplement: Supplementary file 2 — Additional file 2: Table S2. Unit counts and cost (2018 USD) for patient-specific hepatitis A items. [file 12879_2021_6993_MOESM2_ESM.docx]

| **Table S2: Unit counts and cost (2018 USD) for patient-specific hepatitis A items** | | | | | |
| --- | --- | --- | --- | --- | --- |
| **Blood tests** | | | | | |
| **Test** | **Unit counts for adult hepatitis A patient population** | **Unit counts for paediatric hepatitis A patient population** | **Unit cost in USD** | **Total cost USD for adult hepatitis A patient population** | **Total cost USD for paediatric hepatitis A patient population** |
| HAV IgG | 9 | 3 | 8.96 | 80.65 | 26.88 |
| HAV IgM | 219 | 224 | 8.96 | 1962.54 | 2007.34 |
| HBsAg | 128 | 105 | 8.96 | 1147.05 | 940.94 |
| HBC | 63 | 62 | 8.96 | 564.57 | 555.60 |
| HCV | 94 | 82 | 8.96 | 842.37 | 734.83 |
| ALT | 358 | 288 | 3.23 | 1157.74 | 931.36 |
| AST | 306 | 234 | 3.23 | 989.57 | 756.73 |
| ALP | 309 | 214 | 3.08 | 951.93 | 659.27 |
| Albumin | 201 | 145 | 2.86 | 575.61 | 415.24 |
| Total bilirubin | 325 | 243 | 2.51 | 816.14 | 610.22 |
| Conjugated bilirubin | 275 | 208 | 1.91 | 525.95 | 397.81 |
| GGT | 299 | 206 | 3.23 | 966.94 | 666.18 |
| Fibrinogen | 23 | 41 | 2.43 | 56.00 | 99.82 |
| INR | 273 | 247 | 3.37 | 919.50 | 831.93 |
| HB | 230 | 199 | 1.28 | 295.18 | 255.39 |
| FBC Differential Count | 229 | 192 | 2.26 | 518.55 | 434.77 |
| Full Blood Count Incl Platelet | 222 | 190 | 4.13 | 916.75 | 784.60 |
| Neutrophils | 37 | 43 | 2.26 | 83.78 | 97.37 |
| Na | 197 | 166 | 2.16 | 425.65 | 358.67 |
| K+ | 207 | 167 | 2.16 | 447.26 | 360.83 |
| Urea | 191 | 161 | 2.16 | 412.69 | 347.87 |
| Creatine | 224 | 184 | 2.16 | 483.99 | 397.56 |
| HIV | 57 | 67 | 3.93 | 223.90 | 263.19 |
| **Radiology** | | | | | |
| **Test** | **Unit counts for adult hepatitis A patient population** | **Unit counts for paediatric hepatitis A patient population** | **Unit cost in USD** | **Total cost USD for adult hepatitis A patient population** | **Total cost USD for paediatric hepatitis A patient population** |
| AXR | 5 | 2 | 4.47 | 22.37 | 8.95 |
| Abdominal ultrasound | 50 | 36 | 12.68 | 633.90 | 456.41 |
| Liver ultrasound | 1 | 1 | 7.86 | 7.86 | 7.86 |
| Gastroscopy | 1 | 1 | 74.92 | 74.92 | 74.92 |
| Brain CT | 4 | 0 | 60.75 | 242.98 | 0.00 |
| **Medicines and products** | | | | | |
| **Medicines and products** | **Number of prescriptions in adult hepatitis A patient population** | **Number of prescriptions in paediatric hepatitis A patient population** | **Mean unit cost USD per prescription** | **Total cost USD for prescriptions in adult hepatitis A population** | **Total cost USD for prescriptions in paediatric hepatitis A population** |
| Antibiotics | 27 | 31 | 5.33 | 1524.56 | 3019.70 |
| Antifungals | 11 | 5 | 0.79 | 112.13 | 74.75 |
| Antiemetics | 41 | 2 | 0.97 | 38.56 | 575.28 |
| Lactulose | 14 | 3 | 1.31 | 251.08 | 77.51 |
| Steroid | 2 | 2 | 4.71 | 75.84 | 202.22 |
| Vitamin K | 33 | 24 | 2.97 | 1770.66 | 729.50 |
| Other medicines | 128 | 53 | 0.98 | 1841.09 | 785.13 |
| Fresh frozen plasma | 1 | 0 | 157.79 | 2327.44 | NA |
| Platelets | 2 | 0 | 618.97 | 18247.26 | NA |
| Prescriptions at discharge | 145 | 84 | 13.52 | 2149.74 | 946.64 |
